# Assessment of attitude towards COVID-19 vaccine and associated factors among clinical practitioners in Ethiopia: A cross-sectional study

**DOI:** 10.1371/journal.pone.0269923

**Published:** 2022-06-16

**Authors:** Eleleta Surafel Abay, Mezmur Dawit Belew, Beza Seleshi Ketsela, Enderas Eneyew Mengistu, Liya Sisay Getachew, Yonas Ademe Teferi, Abebe Bekele Zerihun

**Affiliations:** 1 Division of COVID-19 Response Task Force, Department of Emergency Medicine and Critical Care, Kadisco General Hospital, Addis Ababa, Ethiopia; 2 Department of Molecular and Computational Biology, University of Southern California, Los Angeles, California, United States of America; 3 Department of Bioengineering, San Diego State University, San Diego, California, United States of America; 4 Department of Outpatient Care, Landmark General Hospital, Addis Ababa, Ethiopia; 5 Department of Clinical Trials, Armauer Hansen Research Institute, Addis Ababa, Ethiopia; 6 Division of Cardiothoracic Surgery, Department of Surgery, Addis Ababa University, Addis Ababa, Ethiopia; Qazvin University of Medical Sciences, ISLAMIC REPUBLIC OF IRAN

## Abstract

**Background:**

Clinical practitioners are influential figures in the public’s health-seeking behavior. Therefore, understanding their attitudes toward the COVID-19 vaccine is critical for implementing successful vaccination programs. Our study aimed to investigate clinical practitioners’ acceptance of the COVID-19 vaccine and associated factors for evidence-based interventions.

**Methods:**

Data from 461 clinical practitioners were collected using a cross-sectional design via an online self-administered survey. Descriptive and multiple logistic regression analyses and chi-square tests were conducted using R version 3.6.1.

**Results:**

The COVID-19 vaccine was accepted by 84.4 percent of those polled, and 86.1 percent said they would recommend it to others. Individuals with advanced levels of education demonstrated greater readiness for vaccine acceptance (P<0.001) and willingness to recommend (P<0.001). On the other hand, practitioners with concerns about the safety of vaccines developed in emergency settings were less likely to accept vaccines (OR = 0.22). Practitioners influenced by social media posts (OR = 0.91) and religious beliefs (OR = 0.71) were found to be less willing to recommend the vaccine.

**Conclusion:**

The study demonstrated that interventions to improve clinical practitioners’ acceptance and recommendation of the COVID-19 vaccine should consider the following factors: level of experience and education, religious beliefs, safety concerns, specific profession, and source of information. Vaccine literacy efforts that directly address specific concerns and misconceptions, such as those that reconcile social media information and religious beliefs with scientific literature, are recommended.

## Introduction

Coronavirus Disease 2019 (COVID-19) is a highly contagious viral infection caused by Severe Acute Respiratory Syndrome Coronavirus 2 (SARS-CoV-2) [1, 2]. COVID-19 was first identified in a cluster of cases in Wuhan, China, on December 31, 2019. The World Health Organization (WHO) later declared it a pandemic on March 11, 2020 [3]. As of March 29, 2021, there are 128 million cases and 2.8 million deaths globally, with 202,545 cases and 2,825 deaths in Ethiopia [4].

The WHO has proposed several public health measures to control the rapid spread of the virus [5], one of which is the rollout of vaccines. Vaccines represent one of the most successful and cost-effective methods of achieving individual and herd immunity [6, 7]. Previous outbreaks have taught us that herd immunity is pathogen-specific [6, 7]. It is estimated that a threshold value of around 67 percent will be sufficient to achieve herd immunity against SARS-CoV-2 [8]. This assumes that the virus’s basic reproductive number (R0) is three, implying that one infected individual infects three new individuals [8].

In response to the virus’s rapid spread, vaccines against COVID-19 were developed in a relatively short period. As of February 18, 2021, at least seven different vaccines had been distributed globally [9]. In vaccine prioritization, the overall rollout strategy recommendations are grounded in the WHO Prioritization Roadmap and Values Framework. These recommendations depend on epidemiologic setting and vaccine supply scenarios [10]. When vaccine supplies are severely constrained, as in the case of Ethiopia, initial focus on covering groups that have been placed at disproportionate risk is justified [10]. In that regard, health workers are prioritized given their high risk of becoming infected and transmitting COVID-19. In addition, their direct interaction with health systems are expected to facilitate effective deployment of vaccine program to other epidemiologic groups [10].

Vaccine hesitancy is the delay in acceptance, or refusal of vaccination, despite the availability of vaccination services [11]. It has been identified by WHO as one of the top ten global health threats in 2019 [12]. Since the onset of the pandemic, several studies conducted both globally and locally have revealed a generally negative attitude toward COVID-19 vaccines in the general population [13, 14]. Existing evidence shows that community confidence in, and acceptance of, vaccines depends on trust in healthcare professionals, healthcare system, science, and socio-political context [15]. Early studies on healthcare worker attitudes reported that overall willingness to get vaccinated lies between 40–90 percent among physicians and nurses [16–21]. Clinical practitioners’ influential position in society and the healthcare system necessitates an assessment of their attitude toward and acceptance of the COVID-19 vaccine. As such, mass vaccination programs may suffer if there is widespread skepticism, low levels of acceptance, and recommendation among healthcare workers, particularly those in clinical practice, who are more likely to influence public attitudes, beliefs, and intentions through direct patient care [22].

Hesitancy and low levels of acceptance and recommendation among clinical practitioners have been linked with the guarantee of safety, hypothetical risk of vaccine and media influence, a lack of trust in health ministries in ensuring vaccine safety and proper delivery of vaccine information [18–20], concerns about efficacy, the speed of vaccine development, the potential for political influence on vaccine development and review processes, and the need for more information [21, 22]. Furthermore, disparities in hospital roles, personal stances, and research and field experts as influential sources of vaccine guidance have been linked to COVID-19 vaccine acceptance among healthcare workers and clinical practitioners [21, 22].

Additionally, several theories on the intention of, and willingness towards, vaccine uptake have been studied and proposed, particularly in line with behavioral proposition [23–30]. Such explanations, that have extensively explored protection motivation, perceived vulnerability, health belief and planned behavior theories, have provided valuable framework for accumulating evidence for health related behavior [24, 25, 27, 29, 30]. In that regard, attitude towards vaccine uptake has been linked to perceived disease and vaccine knowledge with proper acquisition of substantial accurate information found to influence and strengthen adaptive response towards protective health behavior i.e. individual vaccine uptake and public recommendation [23, 24, 27–29]. Personal attitudes, subjective norms, and perceived vulnerability have also been linked to behavioral intention with regards to vaccine uptake [24, 25, 27, 29, 30]. When compared to contextual factors (availability) and vaccine characteristics, individual factors such as health literacy have been found to be more likely altered in health promotion strategies [28]. Clinical practitioners’ attitudes toward vaccines are frequently misunderstood as positive because they have substantial health literacy through extensive scientific and medical knowledge and training [19, 28]. However, clinical practitioners are not uniformly homogeneous in their level of expertise, and thus the majority are not experts in immunology and vaccination [19, 28]. Numerous studies have indicated that vaccine hesitancy exists among clinical practitioners at the varying prevalence and intensity levels that are inversely related to their level of immunology training [19, 21, 22]. Furthermore, vaccine uptake has also been explored in relation to systemic underestimation in standard surveys that fail to provide accurate information on vaccines and herd immunity [23].

At the time of our study, few papers were published assessing clinical practitioners’ acceptance of the vaccine, even fewer on willingness to recommend the vaccine to the public globally, and none in Ethiopia. As of March 29, 2021, the exact number of clinical practitioners who have received vaccination in Ethiopia has not been made public. To the best of our knowledge, this is the first study of its kind to investigate the extent of vaccine acceptance among clinical practitioners in Ethiopia, the reasons for their reluctance, and their willingness to recommend the vaccine. Our study also looked at the relationship between vaccine acceptance and willingness to recommend it and gender, age, the field of work (department of employment), years in practice, religion, and level of education, place of work, vaccination status, history of COVID-19 infection, and direct patient care.

## Materials and methods

### Ethical approval

The Research and Ethics Committee approved the study of Addis Ababa University’s Department of Surgery. The study’s online participation was completely voluntary. Written consent was obtained from participants in the following manner: the first page of the survey provided the "I consent to participate in this survey" segment as a required field. This means that unless participants agree to participate/provide consent at this stage, they will not proceed to the survey questions. Prior to obtaining ethical clearance and approval, this procedure was communicated with the Research and Ethical Committee. Furthermore, the first page included an explanation of the study’s objective and purpose and the authority and ethical clearance under which the study was to be conducted. There was no collection of personally identifiable information or IP addresses from respondents or their devices. All digital data was stored on the password-protected laptop to which only investigators had access.

### Study design and setting

From April to May 2021, a cross-sectional study was conducted in Ethiopia to collect information from clinical practitioners.

### Study sample

The sample consisted of clinical practitioners (physicians, nurses, and health officers) from various specialties working in different health facilities. There are over 70,000 such practitioners in Ethiopia [31]. The country has a 20% internet accessibility/penetration rate [32]. The sample size was estimated to be around 500, assuming that 18% of clinical practitioners with internet access could be reached with a 20% response rate. A total of 464 clinical practitioners clicked on the survey link and agreed to participate. Three of these people did not complete the survey, resulting in 461 responses and an 18.3 percent response rate.

### Study instrument

A self-administered 24-item online survey with four sections was used to collect data. Section one [Table A in S1 Table] gathered primary demographic data. Section two [Table B in S1 Table] included four "yes" or "no" questions assessing attitudes of clinical practitioners toward the COVID-19 vaccine. Section three [Table C in S1 Table] had five questions designed to evaluate their acceptance of the COVID-19 vaccine. Section four [Table D in S1 Table] included five questions assessing their advocacy for the vaccine. Three of these questions used 4-point Likert-scale statements with options of "strongly agree," "agree," "disagree," and "strongly disagree". The survey was based on WHO COVID-19 vaccine information [33] and vaccine research from other countries [19, 34], but it was tailored during the development and pretesting processes to meet the needs of Ethiopian practitioners.

The survey was first developed in English and was later translated into Amharic, Ethiopia’s official professional language, by investigators who are fluent in both languages. During the pretesting process, both translations were tested for linguistic validity by external language experts. The instrument was also pretested for length and scientific clarity by 15 clinical practitioners, purposefully sampled to include infectious disease and public health specialists. The findings and observations made during the pretesting were used to modify the questions and subsequent data collection process.

### Data collection method

Data were collected anonymously through an online survey (Google Forms) between April 22 and May 14, 2021. The survey link was distributed via medical association mailing lists and social media groups in a snowball effect. The inclusion and exclusion criteria for this study’s participants were based on their clinical practice and direct patient contact at the study time. To ensure adherence to this inclusion criteria, the first page included an "I am currently in clinical practice" as a required field before allowing participants to proceed to the survey questions, in addition to strictly sharing the survey link with medical association groups and individuals in practice. Participants in the survey who were not in clinical practice (direct patient care) at the study time were not included.

Additionally, the first page of the survey also included consent to participate as a required field before directing participants to the survey questions. All data from participants was kept confidential by maintaining the anonymity of study respondents.

### Data management and analysis

Demographic data were subjected to descriptive analyses. The Chi-square test was used to assess the distribution of vaccine acceptance and willingness to recommend among our various demographic groups. Multiple logistic regression analyses examined the relationship between attitude, acceptance, and advocacy and various questionnaire factors. All analyses were carried out in R version 3.6.1., with the statistical significance P-value set at 0.05.

## Results

### Socio-demographic data of respondents

We conducted a survey of health care workers in Ethiopia to assess their attitudes toward the COVID-19 vaccine for this study. When we looked at the demographic distribution of the people surveyed, we discovered more male (59.2%) survey respondents than female (40.8%). We also found that most of our participants (68.0%) live with other people, making the study of their attitudes toward the vaccine important because it directly affects multiple people who cohabit with them. Most study participants (63.6%) have advanced degrees, work as physicians (70.7%), and work in government facilities (77.3%).

Our respondents are mostly younger (51.8% are under the age of 30) and consequently have only been in their respective professions for a short period (54.7% have less than five years of experience). Table 1 contains more detailed demographic information on our study’s participants.

**Table 1:** Demographic groups of our survey participants for the study.

Group		Number of survey respondents	Group		Number of survey respondents
**Gender**	Male	270 (59.2%)	**Years of experience**	<5 years	249 (54.7%)
	Female	186 (40.8%)		5–10 years	118 (25.9%)
**Age**	20–29	229 (51.8%)		>10 years	88 (19.4%)
	30–49	202 (45.7%)	**Place of work**	Government Facility	347 (77.3%)
	50+	11 (2.5%)		Private Facility	102 (22.7%)
**Marital status**	Single	292 (63.3%)	**Type of facility**	General Hospital	119 (27.0%)
	Married/Co-habiting	151 (32.8%)		Referral Hospital	205 (46.5%)
	Divorced/Widowed	18 (3.9%)		Primary Hospital	19 (4.3%)
**Living Situation**	Living alone	147 (32.0%)		Private Hospital/Clinic	48 (10.9%)
	Living with family/others	313 (68.0%)		Health Center	50 (11.3%)
**Religion**	Orthodox Christian	264 (57.4%)	**Profession**	Specialist	103 (22.6%)
	Protestant Christian	96 (20.9%)		General Practitioner	139 (30.5%)
	Catholic Christian	10 (2.2%)		Intern	25 (5.5%)
	Muslim	48 (10.4%)		Resident	55 (12.1%)
	Jehovah’s Witnesses	7 (1.5%)		Health Officer	37 (8.1%)
	Other	35 (7.6%)		Nursing Practitioner	96 (21.2%)
**Highest Level of Education Attained**	Bachelor’s Degree	122 (27.1%)			
	Master’s Degree	42 (9.3%)			
	Advanced Degree (MD, PhD)	287 (63.6%)			

### Association between demographics and attitude towards the COVID-19 vaccine

After a good understanding of our survey respondents’ demographics, we set out to investigate their attitudes toward the COVID-19 vaccine. Two questions about our participants’ vaccination status and plans to get vaccinated were used to score the level of vaccine (Table B in S1 Table). If they answered yes to either question, they were labeled as "accepting" the vaccine because it means they have been vaccinated or intend to be. They were classified as "non-accepting" for the COVID-19 vaccine if they answered "No" to both questions.

Similarly, we used survey responses to three questions to score respondents’ willingness to recommend vaccines (Table D in S1 Table). Individuals who responded "strongly agree" to any of the questions received a score of 4. A response of "agree" received a 3, a response of "disagree" received a 2, and response of "strongly disagree" received a 1. As a result, an individual could receive anywhere from 3 to 12 points for each of the three questions. After total scores were computed, all respondents with total scores of 7 or higher were classified as "willing" while those with total scores of less than seven were classified as "unwilling" to recommend the vaccine.

We discovered that vaccine acceptance was high among our survey respondents, with 84.4 percent reporting that they have been vaccinated or intend to be vaccinated in the future. We then wanted to see if there was a link between a person’s demographic group and their willingness to accept the vaccine. As shown in Table 2, we found that level of education (P<0.001) influenced vaccine acceptance, with a higher proportion of vaccine accepting practitioners having a graduate degree (i.e., MD or Ph.D.). Vaccine acceptance was also affected by the type of facility (P<0.001), with health centers having a lower proportion of vaccine accepting practitioners than other facilities. Furthermore, vaccine acceptance was influenced by the professions of our respondents (P<0.001), with health officers having more individuals who were vaccine non-accepting than those who were vaccine-accepting.

**Table 2:** Association data regarding the vaccine acceptance of the different demographic groups of our survey participants.

Group		Vaccine Accepting	Vaccine Non-Accepting	P-Value from X^2^ test
**Gender**	Male	225 (49.3%)	45 (9.9%)	0.52
	Female	160 (35.1%)	26 (5.7%)	
**Age**	20–29	193 (43.7%)	36 (8.1%)	0.84
	30–49	171 (38.7%)	31 (7.0%)	
	50+	10 (2.3%)	1 (0.2%)	
**Marital status**	Single	245 (53.1%)	47 (10.2%)	0.26
	Married/Co-habiting	131 (28.4%)	20 (4.3%)	
	Divorced/Widowed	13 (2.8%)	5 (1.1%)	
**Living Situation**	Living alone	123 (26.7%)	24 (5.2%)	0.89
	Living with family/others	265 (57.7%)	48 (10.4%)	
**Religion**	Orthodox Christian	217 (47.2%)	47 (10.2%)	0.08
	Protestant Christian	83 (18.0%)	13 (2.8%)	
	Catholic Christian	10 (2.2%)	0 (0.0%)	
	Muslim	41 (8.9%)	7 (1.5%)	
	Jehovah’s Witnesses	4 (0.9%)	3 (0.7%)	
	Other	33 (7.2%)	2 (0.4%)	
**Highest Level of Education Attained**	Bachelor’s Degree	89 (19.7%)	33 (7.3%)	<0.001
	Master’s Degree	35 (7.8%)	7 (1.6%)	
	Advanced Degree (MD, PhD)	257 (57.0%)	30 (6.6%)	
**Years of experience**	<5 years	211 (46.4%)	38 (8.4%)	0.18
	5–10 years	106 (23.3%)	12 (2.6%)	
	>10 years	71 (15.6%)	17 (3.7%)	
**Place of work**	Government Facility	292 (65.0%)	55 (12.2%)	0.71
	Private Facility	88 (19.6%)	14 (3.2%)	
**Type of facility**	General Hospital	107 (24.3%)	12 (2.7%)	<0.001
	Referral Hospital	183 (41.5%)	22 (4.9%)	
	Primary Hospital	14 (3.2%)	5 (1.1%)	
	Private Hospital/Clinic	41 (9.3%)	7 (1.6%)	
	Health Center	29 (6.6%)	21 (4.8%)	
**Profession**	Specialist	100 (22.0%)	3 (0.7%)	<0.001
	General Practitioner	116 (25.5%)	23 (5.1%)	
	Intern	17 (3.7%)	8 (1.8%)	
	Resident	53 (11.6%)	2 (0.4%)	
	Health Officer	17 (3.7%)	20 (4.4%)	
	Nursing Practitioner	83 (18.2%)	13 (2.9%)	

Similar to vaccine acceptance, the general tendency of individuals’ willingness to recommend the vaccine was high, with 86.1% of our participants falling into this category. Furthermore, our study revealed that religious background influenced willingness to recommend the vaccine (P = 0.02), with practitioners in the "Jehovah’s Witness" category more reluctant to recommend it than other cohorts. Nonetheless, we believe it is important to highlight our study’s disproportionately small "Jehovah’s Witness" sample size. We also discovered that practitioners’ willingness to recommend the vaccine was affected by their level of education (P = 0.01), with advanced degrees closely associated with an individual readily recommending the vaccine to others. Additionally, our study identified that a person’s profession (P<0.001), years of experience (P = 0.01), and the type of facility they work in (P<0.01) all have a significant impact on their willingness to recommend the COVID-19 vaccine (Table 3).

**Table 3:** Association data between the willingness to recommend the vaccine and the different demographic groups of our survey participants.

Group		Willing to recommend vaccine	Unwilling to recommend vaccine	P-Value from X^2^ test
**Gender**	Male	228 (50.0%)	42 (9.2%)	0.32
	Female	164 (36.0%)	22 (4.8%)	
**Age**	20–29	200 (45.2%)	29 (6.6%)	0.67
	30–49	171 (38.7%)	31 (7.0%)	
	50+	9 (2.0%)	2 (0.5%)	
**Marital status**	Single	251 (54.4%)	41 (8.9%)	0.18
	Married/Co-habiting	133 (28.9%)	18 (3.9%)	
	Divorced/Widowed	13 (2.8%)	5 (1.1%)	
**Living Situation**	Living alone	124 (27.0%)	23 (5.0%)	0.55
	Living with family/others	272 (59.1%)	41 (8.9%)	
**Religion**	Orthodox Christian	229 (49.8%)	35 (7.6%)	0.02
	Protestant Christian	83 (18.0%)	13 (2.8%)	
	Catholic Christian	10 (2.2%)	0 (0.0%)	
	Muslim	40 (8.7%)	8 (1.7%)	
	Jehovah’s Witnesses	3 (0.7%)	4 (0.9%)	
	Other	31 (6.7%)	4 (0.9%)	
**Highest Level of Education Attained**	Bachelor’s Degree	86 (9.5%)	36 (4.0%)	<0.001
	Master’s Degree	35 (3.9%)	7 (0.8%)	
	Advanced Degree (MD, PhD)	267 (29.5%)	20 (2.2%)	
**Years of experience**	<5 years	217 (24.0%)	32 (3.5%)	0.01
	5–10 years	108 (11.9%)	10 (1.0%)	
	>10 years	68 (7.5%)	20 (2.2%)	
**Place of work**	Government Facility	294 (65.5%)	53 (11.8%)	0.22
	Private Facility	92 (20.5%)	10 (2.2%)	
**Type of facility**	General Hospital	104 (23.6%)	15 (3.3%)	<0.001
	Referral Hospital	189 (42.9%)	16 (3.6%)	
	Primary Hospital	16 (3.6%)	3 (0.7%)	
	Private Hospital/Clinic	45 (10.2%)	3 (0.7%)	
	Health Center	25 (5.7%)	25 (5.7%)	
**Profession**	Specialist	101 (22.2%)	2 (0.4%)	<0.001
	General Practitioner	124 (27.3%)	15 (3.3%)	
	Intern	18 (4.0%)	7 (1.5%)	
	Resident	54 (11.9%)	1 (0.2%)	
	Health Officer	15 (3.3%)	22 (4.8%)	
	Nursing Practitioner	81 (17.8%)	15 (3.3%)	

### Other factors that affect attitude towards the COVID-19 vaccine

We then wanted to see if any other factors influenced healthcare practitioners’ attitudes toward the COVID-19 vaccine. For this analysis, the following factors were investigated: (1) having screened/treated known COVID-19 cases, (2) being diagnosed with COVID-19, (3) knowing someone who had a strong reaction to the COVID-19 vaccine, (4) knowing someone who refused to take the COVID-19 vaccine, (5) opinion on acquiring immunity through vaccination and (6) opinion on the safety of vaccine developed in an emergency setting.

We found that strong disagreement about acquiring immunity naturally rather than through vaccines positively influenced an individual’s vaccine acceptance (OR = 10.90; 95% CI = 2.82–73.36). Furthermore, screening or treating a known COVID-19 case increased vaccine acceptance (OR = 4.02; 95% CI = 2.08–7.93). Concerns about the safety of vaccines developed in an emergency setting, on the other hand, influenced our respondents’ acceptance of the COVID-19 vaccine (OR = 0.22; 95% CI = 0.11–0.46). Surprisingly, having been diagnosed with COVID-19 or knowing someone who had a severe reaction to the vaccine did not affect our respondents’ willingness to accept the vaccine (OR = 0.96; 95% CI = 0.48–2.01). Table 4 shows the effect of each factor investigated in this study on our participants’ vaccine acceptance.

**Table 4:** Effects of non-demographic factors on vaccine acceptance of our survey respondents.

Possible factors affecting a respondent’s COVID-19 vaccine acceptance	Odds Ratio	95% CI
Having screened and/or treated any known COVID-19 patient	4.02	2.08–7.93
Having been diagnosed with COVID-19 in the past	0.96	0.48–2.01
**Disagreement** to the statement–It is better to acquire immunity to infectious diseases naturally	4.68	2.30–9.84
**Strong agreement** to the statement–It is better to acquire immunity to infectious diseases naturally	3.29	1.23–9.55
**Strong disagreement** to the statement–It is better to acquire immunity to infectious diseases naturally	10.90	2.82–73.36
Knowing someone who has had a serious reaction to the vaccine	0.54	0.28–1.04
**Disagreement** to the statement—The safety of vaccines developed in an emergency cannot be guaranteed	3.05	1.07–11.00
**Strong agreement** to the statement—The safety of vaccines developed in an emergency cannot be guaranteed	0.22	0.11–0.46
**Strong disagreement** to the statement—The safety of vaccines developed in an emergency cannot be guaranteed	1.69	0.22–37.12
Knowing anyone who has refused to take the COVID-19 vaccine	0.26	0.04–1.01

Our study also revealed that strong disagreement to acquiring immunity through natural means rather than vaccines (OR = 20.14; 95% CI = 3.90–370.00) and having screened or treated any known COVID-19 patients (OR = 3.53; 95% CI = 1.77–7.17) had a positive effect on a practitioner’s willingness to recommend the vaccine while having treated a known COVID-19 case and knowing someone who had a severe reaction to the vaccine had a negative effect on a practitioner’s willingness to recommend the vaccine (OR = 0.41; 95% CI = 0.21–0.81). The combined effect of these factors on our respondents’ willingness to recommend the vaccine is shown in Table 5.

**Table 5:** Effects of non-demographic factors on our survey respondents’ willingness to recommend the COVID-19 vaccine.

Possible factors affecting a respondent’s willingness to recommend the COVID-19 vaccine	Odds Ratio	95% CI
Having screened and/or treated any known COVID-19 patient	3.53	1.77–7.17
Having been diagnosed with COVID-19 in the past	1.19	0.56–2.62
**Disagreement** to the statement–It is better to acquire immunity to infectious diseases naturally	6.53	3.09–14.64
**Strong agreement** to the statement–It is better to acquire immunity to infectious diseases naturally	3.18	1.16–9.68
**Strong disagreement** to the statement–It is better to acquire immunity to infectious diseases naturally	20.14	3.90–370.00
Knowing someone who has had a serious reaction to the vaccine	0.41	0.21–0.81
**Disagreement** to the statement—The safety of vaccines developed in an emergency cannot be guaranteed	2.71	0.93–9.90
**Strong agreement** to the statement—The safety of vaccines developed in an emergency cannot be guaranteed	0.49	0.23–1.07
**Strong disagreement** to the statement—The safety of vaccines developed in an emergency cannot be guaranteed	7.67 x 10^6^	1.16x10^-10^–1.55x10^155^
Knowing anyone who has refused to take the COVID-19 vaccine	0.36	0.05–1.44

### Sources of COVID-19 information and attitudes towards the vaccine

We found that published research is the most popular source of information (n = 271, 58.8%). This is followed by the views of leading researchers and public health and infectious disease specialists (n = 211, 45.8%), Ministry of Health announcements (n = 204, 44.2%), social media posts (n = 96, 20.8%), and opinions from religious leaders (n = 46, 9.9%). With the exception of social media, all information sources had a significant impact on vaccine acceptance. Information based on religion and religious leaders (OR = 0.15; 95% CI = 0.67–0.84) correlated negatively with vaccine acceptance (Table 6). On the other hand, all sources of information showed a significant correlation with the willingness to recommend the vaccine. A negative correlation was found between information based on social media posts (OR = 0.91; 95% CI = 0.84–0.98) and religion or religious leaders (OR = 0.71; 95% CI = 0.65–0.79). (Table 7).

**Table 6:** Relationship between our participants’ source of information regarding COVID-19 and their acceptance of its vaccine.

Source of information regarding COVID-19 and its vaccine	Odds Ratio	95% CI
Published research	1.16	1.08–1.23
Social media posts	0.94	0.87–1.03
Ministry of Health	1.20	1.13–1.28
Leading researchers and public health and infectious disease specialists	1.08	1.01–1.15
Religion and religious leaders	0.15	0.67–0.84

**Table 7:** Determining the relationship between our participants’ source of information regarding the COVID-19 vaccine and their willingness to recommend the vaccine.

Source of information regarding COVID-19 and its vaccine	Odds Ratio	95% CI
Published research	1.20	1.13–1.27
Social media posts	0.91	0.84–0.98
Ministry of Health	1.15	1.09–1.22
Leading researchers and public health and infectious disease specialists	1.10	1.04–1.16
Religion and religious leaders	0.71	0.65–0.79

## Discussion

As of May 26, 2021, Ethiopia had vaccinated a total of 1,738,550 people. Data on the number of clinical practitioners immunized in the country is still outstanding. This is the first study to assess clinical practitioners’ attitudes toward the COVID-19 vaccine and associated factors to the best of our knowledge. Their ability to influence health behavior necessitates the use of evidence-based knowledge to guide public acceptance of the vaccine and ensure universal coverage.

In addition to social distancing measures, rigorous vaccine administration and coverage are essential in the global fight against the pandemic. As mass coverage weapons, vaccines are the most effective strategy for countries like Ethiopia, with limited medical supplies [35] and a low healthcare provider-to-population ratio [36]. Our study reflected that clinical practitioners are critical for increased public vaccine acceptance and mass coverage.

During the time of this study, AstraZeneca/Oxford vaccine was the only available vaccine in Ethiopia. With a 63.09% efficacy, the vaccine was made available for use in low- and middle-income countries due to easy storage requirements [37] The AstraZeneca/Oxford mass vaccination campaign was launched in the country on March 13, 2021 [38]. On March 15, 2021 several European counties suspended the use of the vaccine following concerns over potential link of the vaccine with Cerebral Venous Thrombosis (CVT) [26, 39]. An investigation conducted by the European Medicine Agency (EMA) found no clear evidence that could confirm the link between AstraZeneca/Oxford vaccine and increased risk of CVT [40]. A comparison was also made between vaccine risk and benefits, which found 22 thrombotic events out of the 3 million people who received that AstraZeneca/Oxford vaccine at the time [41]. It was additionally concluded by EMA that even if CVT risk was in fact confirmed, the health benefits of the vaccine would still significantly outweigh the risks [41]. This was further backed by a research by the University of Oxford which found that the risk of developing CVT from COVID-19 (39 per million) is roughly 8 times higher than the risk of CVT from the AstraZeneca/Oxford Vaccine (5 per million) [42]. Despite such findings, initial vaccine suspension was evidenced through a rise in vaccine hesitancy across Europe [43].

In line with these findings, and the time sensitive need to develop herd immunity against COVID-19, our study has attempted to analyze the attitude of clinical practitioners against the vaccine as first recipients in Ethiopia. Being vaccinated and remaining unvaccinated both involve risks. Such stances must be carefully studied among clinical practitioners particularly as they relate to their willingness to recommend use to the public, as it is within their professional duty to protect their patients and the community at large from undue health risks. Clinical research and data are not easy to comprehend, particularly for the larger public. It is especially difficult in highly uncertain situations where there aren’t timely scientific references to help determine if risks are worth taking [26]. Therefore, it remains up to clinical practitioners to relay scientific vaccine information and reconcile hesitancy in that regard.

According to the study findings, acceptance and willingness to recommend were high at 84.4% and 86.1%, respectively. The acceptability of the COVID-19 vaccine in this study is comparable to the study conducted by Barry M. et al. in Saudi Arabia [44], which found that 70% of healthcare workers accepted the COVID-19 vaccine. Similarly, the acceptability reported in this study is consistent with the multi-country study conducted by Verger P, et al. [19], which reported 72.4% acceptance and 79.6% certain or probable recommendation of the vaccine by healthcare workers. Conversely, the approval of the COVID-19 vaccine in this study is higher than in other countries in the SSA (Sub-Saharan Africa) region. A study [45] conducted in the Democratic Republic of the Congo discovered that approximately 28% of healthcare workers accept the COVID-19 vaccine, while another [46] conducted in Ghana discovered that 39% of healthcare workers accept the vaccine.

In line with the findings of the multi-country study, our research identified safety concerns as a factor influencing practitioners’ acceptance of the vaccine. In addition, our findings paralleled those of Shaw J. et al. [34] in that treatment of a confirmed COVID-19 patient is a factor that influences vaccine acceptance.

The following findings are peculiar to our study: reservations about acquiring immunity through vaccination (as opposed to natural immunity) affect COVID-19 vaccine acceptance, history of COVID-19 infection, or knowledge of someone who has had a severe reaction to the vaccine does not influence vaccine acceptance. Furthermore, our research found that health centers are a breeding ground for practitioners who are equally unwilling to recommend the vaccine to the public (a trend not seen in other practice sites). It also revealed that respondents who work as health officers have lower rates of recommendation willingness. Health officers are the dominant practitioners in health centers and peri-urban and rural areas of the country. As a result, identifying vaccine misconceptions among health officers is critical.

Our study also showed that vaccine acceptance and willingness to recommend are directly related to education level (i.e., as educational level increases, so does the acceptance and the willingness to recommend). This suggests that senior practitioners should be involved in vaccination campaigns. Religion is a factor in vaccine recommendation, despite being reported by a small proportion of our respondents. As such, efforts must be made to increase vaccine literacy through religious reconciliation. The negative correlation between social media posts and willingness to recommend in our study highlights the importance of disseminating vaccine information through posts that link to peer-reviewed research articles.

### Limitations

Our study’s findings must be interpreted in light of the following limitations.

The study used an online data collection tool, as a conventional paper-based survey during the COVID-19 pandemic would violate contact restriction measures. Although the trends in the results provide important insights into attitudes toward the COVID-19 vaccine, direct generalizations to all clinical practitioners in the country are not possible. In rural Ethiopia, digital and internet access is limited. As such, access to vaccine information is limited in these areas. As a result, vaccine acceptance and recommendation levels are expected to be lower than the study results. However, to ensure inclusive representation, measures were taken to reach out to practitioners in rural areas who have digital and internet access to collect and enter responses from those who do not.

It should also be noted that the results exclude data from the Tigray region. Due to ongoing conflict, Internet access in the region was unavailable during the study period. Unfortunately, the magnitude of unrest and communication barriers in the area outweighed the investigators’ efforts to reach practitioners there.

## Conclusion and recommendation

Overall, the results indicate that clinical practitioners accept and are willing to recommend the COVID-19 vaccine. Nonetheless, some factors have a negative correlation with vaccine acceptance and willingness rates. To achieve the desired results, the study demonstrated that interventions to improve the acceptance and recommendation of the COVID-19 vaccine among clinical practitioners must consider the following factors: level of experience and education, religious beliefs, safety concerns, specific profession, and source of information.

It is necessary to identify reasons among practitioners who are more willing to accept and recommend the vaccine and translate them into an actionable strategy to engage the non-willing practitioners. Interventions to increase vaccine literacy and acceptance that address specific concerns and misconceptions are advised. These interventions should be sensitive to religious beliefs, and measures should be taken to disseminate vaccine information that reconciles these beliefs through posts linked to peer-reviewed studies.

## Supporting information

S1 Table Survey questionnaire. (DOCX)

S2 Table Key to column headers to main dataset. (DOCX)

S3 Table Main dataset. (XLSX)

S1 Appendix R script for data analysis. (R)
