# Supplementary material for: Assessment of attitude towards COVID-19 vaccine and associated factors among clinical practitioners in Ethiopia: A cross-sectional study
Source: PLoS One. 2022 Jun 16;17(6):e0269923. doi: 10.1371/journal.pone.0269923 (PMC9202929; doi:10.1371/journal.pone.0269923)
Supplement: S1 Table — (DOCX) [file pone.0269923.s001.docx]

Survey of attitude towards, acceptance of, and advocacy for COVID-19 vaccine (የኮቪድ-19 ክትባት አመለካከት፤ ተቀባይነት እና ድጋፍ የዳሰሳ ጥናት መጠየቂያ ቅጽ)

This survey aims to collect the attitude towards, acceptance of, and advocacy for COVID-19 vaccine among physicians across different specializations, nurses and health officers. By part-taking in this study, you will provide valuable information to help improve existing programs and design better solutions to improve our health system.

This study is conducted through the Addis Ababa University, School of Medicine, Department of Surgery. Ethical clearance to undertake this study has been obtained from the Research and Ethical Committee of the department. The survey will only take approximately 10 minutes to complete. Please be advised that you do not need to provide your name or any personal identifiable information. All information you entered will be confidential. Your participation is completely voluntary and you can stop or skip any questions you do not wish to answer at any time. If you have any questions about this study, please contact us at [yonyademe@gmail.com](mailto:yonyademe@gmail.com)

የዚህ ዳሰሳ ጥናት ዓላማ የጤና ባለሙያ ሠራተኞች (ሃኪሞች ፣ ነርሶች እና የጤና መኮንኖች) ስለ ኮቪድ-19 ክትባት ያላቸውን ግንዛቤ እና ለህብረተሰቡ ያላቸውን ፈቃደኝነት ለመሰብሰብ ነው ፡፡ በዚህ ጥናት ውስጥ በመሳተፍዎ ፣ ፕሮግራሞችን እና የጤና ስርዓታችንን ለማሻሻል የሚረዱ መንገዶችን እና ጠቃሚ መረጃዎችን ለመሰብሰብ ይረዱናል፡፡

ይህ ጥናት የሚካሄደው በአዲስ አበባ ዩኒቨርስቲ፤ የህክምና ትምህርት ቤት የቀዶ ጥገና ክፍል ነው። ይህን የጥናት ዳሰሳ ለማካሄድ ከዚህ ክፍል የስነምግባራዊ ፈቃድ የተገኘ ሲሆን፤ የዳሰሳውን ጥያቄዎች ለማጠናቀቅ 10 ደቂቃ ያህል ብቻ ይበቃል ፡፡ ስምዎን ወይም ማንኛቸውም የግል መለያ መረጃዎችን መስጠት አያስፈልግዎትም ፣ የሚሰጡት መረጃ በሙሉ ሚስጥራዊ ነው ፡፡ ተሳትፎዎ ሙሉ በሙሉ ፈቃደኝነት ላይ የተመሰረተ ነው። በማንኛውም ጊዜ መመለስ የማይፈልጉትን ማንኛውንም ጥያቄ ማቆም ወይም መዝለል ይችላሉ ፡፡ ስለዚህ ጥናት ማንኛቸውም ጥያቄዎች ካሉዎት እባክዎን በ[yonyademe@gmail.com](mailto:yonyademe@gmail.com) ያግኙን ፡፡

I am (tick the following boxes):

እኔ (የሚከተሉትን ሳጥኖች ምልክት ያድርጉ):

- A physician, a nurse, a health officer
  - ሃኪም ፣ ነርስ ፣ የጤና መኮንን ነኝ
- Willing to participate in this survey
  - በዚህ የዳሰሳ ጥናት ለመሳተፍ ፈቃደኛ ነኝ
- Currently in clinical practice
  - በአሁን ስዐት ከታካሚዎች ጋር ቀጥተኛ ንክኪ ያለው የህክምና ስራ ላይ እገኛለሁ

*if both boxes are ticked, participants will be directed to the survey questions.

Table A: Socio-demographic information questions

| **Section 1፡ Socio-demographic Info** | **ክፍል 1፡ የስነ ህዝብ መረጃ** |
| --- | --- |
| Gender (ጾታ) | Male (ወንድ)  Female (ሴት) |
| Age (እድሜ) | _____ |
| Marital status (የጋብቻ ሁኔታ) | Single (ያላገባ/ች)  Married/ co-habitating (ያገባ/ች ወይም አብሮ በመኖር ላይ ያሉ)  Divorced/ widowed (የተፋታ/ች ወይም በሞት የተለዩ) |
| Living situation (የኑሮ ሁኔታ) | Living alone (ለብቻ)  Living with family/others (ከቤተሰብ/ከሌሎች ጋር) |
| Religion (ሃይማኖት) | Muslim (ሙስሊም)  Orthodox Christian (ኦርቶዶክስ ክርንቲያን)  Protestant Christian (ፕሮቴስታንት ክርስቲያን)  Catholic Christian (ካቶሊክ ክርስቲያን)  Jehovah’s Witness (የያህዌ ምስክር)  Other (ሌላ) |
| Level of education  Highest level attained (ያጠናቀቁት ከፍተኛ የትምህርት ደረጃ) | Bachelor’s degree (ዲግሪ)  Master’s degree (ማስተርስ ዲግሪ)  Advanced degree (PhD, MD) (የህክምና ዲግሪ ወይም ፒ.ኤች.ዲ) |
| Place of work (የሚሰሩበት ተቋም ዓይነት) | Private facility (የግል ተቋም)  Government facility (የመንግስት ተቋም) |
| Type of facility (የሚሰሩበት ተቋም ምድብ) | Health post (ጤና ኬላ)  Health center (ጤና ጣቢያ)  Primary hospital (የመጀመርያ ደረጃ ሆስፒታል)  General hospital (አጠቃላይ ሆስፒታል)  Referral hospital (ሪፈራል ሆስፒታል)  Private hospital/clinic (የግል ሆስፒታል ወይም ክሊኒክ) |
| Profession (ሙያ) | Physician (ሃኪም):   - Intern (ተለማማጅ ሃኪም) - General Practitioner (ጠቅላላ ሃኪም) - Specialist (ስፔሻሊስት)   Nurse practitioner (ነርስ)  Health Officer (የጤና መኮንን) |
| Years of practice (በስራ ላይ የቆዩበት ጊዜ) | <5 years (ከ 5 አመት በታች)  5-10 years (ከ 5 እስከ 10 አመት)  >10 years (ከ10 አመት በላይ) |
| Table B: Questions to assess general attitude towards COVID-19 | |
| **Section 2: Attitude towards COVID-19 vaccine** | **ክፍል 2፡ ለኮቪድ-19 ክትባት ያሎት አመለካከት** |
| Have you screened and/or treated any known COVID-19 patient?  (ማንኛውንም የታወቀ የCOVID-19 በሽተኛ መርምረዋል ወይም አክመዋል?) | Yes (አዎ)  No (አይደለም) |
| Have you been vaccinated for COVID-19?  (የCOVID-19 ክትባት ወስደዋል?) | Yes (አዎ)  No (አይደለም) |
| If your answer to the above question is no, do you intend to get vaccinated for COVID-19?  ከላይ ለተጠቅሰው ጥያቄ መልስዎ አይደለም ከሆነ የCOVID-19 ክትባት ለመውሰድ ያስባሉ? | Yes (አዎ)  No (አይደለም) |
| Table C: Questions to assess acceptance of COVID-19 vaccine | |
| **Section 3: Acceptance of COVID-19 vaccine** | **ክፍል 3፡ ለኮቪድ-19 ክትባት ያሎት አቀባበል** |
| In my opinion, it is better to acquire immunity to infectious diseases naturally, rather than through vaccination  በሽታ የመከላከል አቅምን በክትባት ከማካበት ይልቅ በተፈጥሮአዊ መንገድ ማካበት የተሻለ ነው ብዬ አስባለሁ | Strongly agree (በጥብቅ እስማማለሁ)  Agree (እስማማለሁ)  Disagree (አልስማማም)  Strongly disagree (በጥብቅ አልስማማም) |
| In my opinion, the safety of vaccines developed in an emergency cannot be guaranteed  በአስቸኳይ የተመረተ ክትባት አስተማማኝነቱ ያጠራጥረኛል | Strongly agree (በጥብቅ እስማማለሁ)  Agree (እስማማለሁ)  Disagree (አልስማማም)  Strongly disagree (በጥብቅ አልስማማም) |
| If your response to the above question is agree or strongly agree, which of the following concerns you the most  ከላይ ለተጠቅሰው ጥያቄ መልስዎ በጥብቅ እስማማለሁ ወይም እስማማለሁ ከሆነ ከሚከተሉት የትኛው ያሳስቦታል | Efficacy (የመከላከል አቅሙ)  Side effect profile (አሉታዊ መዘዙ)  Research and authorization process (የጥናት እና የፍቃድ ሂደት)  Rushed release (የተፋጠነ አቅርቦት) |
| My decision to receive and/or advocate for the vaccine comes from  ክትባቱን የመቀበል እና የመደገፍ አቋሜ የሚወሰነው | Published research (ለህትመት የበቃ ጥናት)  Social media (ማህነራዊ ገጾች)  Ministry of Health (ጤና ጥበቃ)  Leading researchers, public health and infectious diseases specialists (ተመራማሪዎች ፣ የህብረተሰብ ጤና እና ተላላፊ በሽታዎች ባለሙያዎች)  Religion and religious leaders (ሃይማኖት እና የሃይማኖት መሪዎች) |
| Table D: Questions to assess advocacy for COVID-19 vaccine | |
| **Section 4: Advocacy for COVID-19 vaccine** | **ክፍል 4፡ ለኮቪድ-19 ክትባት ያሎት ድጋፍ** |
| I am willing to recommend COVID-19 vaccination to my patients  የCOVID-19 ክትባትን ታካሚዎቼ እንዲወስዱ አበረታታለሁ | Strongly agree (በጥብቅ እስማማለሁ)  Agree (እስማማለሁ)  Disagree (አልስማማም)  Strongly disagree (በጥብቅ አልስማማም) |
| I am willing to recommend COVID-19 vaccination to members of my family  የCOVID-19 ክትባትን የቤተሰብ አባሎቼ እንዲወስዱ አበረታታለሁ | Strongly agree (በጥብቅ እስማማለሁ)  Agree (እስማማለሁ)  Disagree (አልስማማም)  Strongly disagree (በጥብቅ አልስማማም) |
| I am willing to recommend COVID-19 vaccination to people in my community  የCOVID-19 ክትባትን በአካባቢዬ ያሉ ነዋሪዎች እንዲወስዱ አበረታታለሁ | Strongly agree (በጥብቅ እስማማለሁ)  Agree (እስማማለሁ)  Disagree (አልስማማም)  Strongly disagree (በጥብቅ አልስማማም) |
