# Supplementary material for: Assessment of attitude towards COVID-19 vaccine and associated factors among clinical practitioners in Ethiopia: A cross-sectional study
Source: PLoS One. 2022 Jun 16;17(6):e0269923. doi: 10.1371/journal.pone.0269923 (PMC9202929; doi:10.1371/journal.pone.0269923)
Supplement: S2 Table — (DOCX) [file pone.0269923.s002.docx]

**Key to Column Headers on the Main Dataset**

| **Q1** | Gender |
| --- | --- |
| **Q2** | Age |
| **Age Groups** | People were grouped into ages 20-29, 30-49 and 50+ based on their age |
| **Q3** | Marital status |
| **Q4** | Living situation |
| **Q5** | Religion |
| **Q6** | Level of education (Highest level attained) |
| **Q7** | Place of work |
| **Q8** | Type of facility |
| **Q9** | Profession |
| **Q10** | Years of practice (including years of residency) |
| **Q11** | Have you screened and/or treated any known COVID-19 patient? |
| **Q12** | Have you been diagnosed with COVID-19 in the past? |
| **Q13** | Have you been vaccinated for COVID-19? |
| **Q14** | If your answer to the above question is no, do you intend to get vaccinated for COVID-19? |
| **VAS** | Vaccine acceptance score. 1 = vaccine accepting individual (answered “Yes” to either Q13 or Q14) ; 0 = vaccine non-accepting individual (answered “No” to both Q13 and Q14) |
| **Q15** | In my opinion, it is better to acquire immunity to infectious diseases naturally, rather than through vaccination |
| **Q16** | Do you personally know anyone who has had a serious reaction to the vaccine? |
| **Q17** | My decision to receive and/or advocate for the vaccine comes from (Select all that apply). 5 columns columns were made for each source of information about COVID-19 and its vaccines. A response of “Y” means that the individual selected that as a source of information for their advocacy for the vaccine and an “N” means they did not. |
| **Q18** | In my opinion, the safety of vaccines developed in an emergency cannot be guaranteed |
| **Q19** | If your response to the above question is agree or strongly agree, which of the following concerns you the most? 4 columns were made for each source of concenrn about the COVID-19 vaccine. A response of “Y” means that the individual selected that as their main source of concern and an “N” means they did not. |
| **Q20** | Do you know anyone who has refused to take the COVID-19 vaccine? |
| **Q21** | If your answer is yes for the above question, How many people? |
| **Q22** | I am willing to recommend COVID-19 vaccination to my patients |
| **Q23** | I am willing to recommend COVID-19 vaccination to members of my family |
| **Q24** | I am willing to recommend COVID-19 vaccination to people in my community |
| **VRS** | Vaccine recommendation score – a score out of 3 - 12. It is a cumulative score for the responses to Q22, Q23 and Q24. (Strongly agree = 4, agree = 3, disagree = 2 and strongly disagree = 1) |
| **VRC** | Categories of individuals based on willingness to recommend the vaccine. W = willing to recommend the vaccine (VRS of 3 – 8); U = unwilling to recommend the vaccine (VRS of 9-12) |
